# Beyond preclinical promise: can mesenchymal stromal cell-derived extracellular vesicles reliably target tubular epithelial cells?

**DOI:** 10.20517/evcna.2025.54

**Published:** 2025-09-23

**Authors:** Linrong Pan, Sergio G. Garcia, Miriam Font-Morón, Marta Sanroque-Muñoz, Marta Clos-Sansalvador, Gisela de Miguel Garcia, Francesc E. Borràs, Marcella Franquesa

**Affiliations:** ^1^REMAR-IGTP Group Germans Trias i Pujol Research Institute (IGTP) and Nephrology Department, University Hospital Germans Trias i Pujol (HUGTiP), Badalona 08916, Spain.; ^2^Department of Cell Biology, Physiology and Immunology Universitat Autònoma de Barcelona (UAB), Bellaterra 08193, Spain.; ^3^Department of Biochemistry and Molecular Biology, Universitat Autònoma de Barcelona (UAB), Bellaterra 08193, Spain.; ^4^Department of Cell Biology, Physiology and Immunology Universitat de Barcelona (UB), Barcelona 08028, Spain.

**Keywords:** Kidney disease, mesenchymal stem/stromal cells, extracellular vesicles, tubular epithelial cells, targeting, biodistribution

## Abstract

Kidney disease, encompassing both acute kidney injury (AKI) and chronic kidney disease (CKD), represents a major global health challenge. A pivotal aspect of the pathogenesis of these conditions is damage to renal tubular epithelial cells (TECs), which contributes to maladaptive repair mechanisms and fibrosis. Due to their essential role, TECs are regarded as a promising target for innovative therapeutic strategies. Mesenchymal stromal cell-derived extracellular vesicles (MSC-EVs) have attracted increasing attention for their therapeutic potential in kidney disease, with extensive literature documenting their beneficial effects on TEC damage through targeted mechanisms. In this review, we critically examine the existing literature on the targeting of TECs by MSC-EVs in both *in vitro* and *in vivo* settings. Furthermore, we highlight the limitations and potential of MSC-EV-based strategies for TEC targeting, aiming to provide insights for future clinical trials and therapeutic applications.

## INTRODUCTION

### Kidney disease and tubular epithelial cell damage

Kidney disease is broadly categorized into two primary forms: acute kidney injury (AKI) and chronic kidney disease (CKD). AKI, marked by damage to renal tubular epithelial cells (TECs), is a common and potentially life-threatening condition among hospitalized patients, affecting 10%-15% of all admissions and approximately 50% of individuals in intensive care units. Severe, recurrent, and inadequately managed AKI can progress to CKD, a process known as the AKI-to-CKD transition^[1]^. CKD is characterized by the gradual loss of kidney function and poses a major global public health challenge^[2]^. According to estimates from the World Health Organization (WHO), CKD accounted for 1.5% of global deaths in 2012 and is projected to become the fifth leading cause of mortality worldwide by 2040^[3]^.

Renal fibrosis, particularly tubulointerstitial fibrosis (TIF), which is marked by tubular atrophy and extracellular matrix (ECM) accumulation^[4]^, is an inevitable outcome of all progressive CKD. Currently, no effective treatment exists to reverse kidney fibrosis and prevent progression to kidney replacement therapy. Among the various renal cell types, TECs are the primary targets of injury. They undergo cell death followed by regeneration and repair^[5]^. However, incomplete recovery can lead to maladaptive repair, fibrosis, and ultimately kidney dysfunction^[6]^. Recent studies have increasingly focused on the mechanisms underlying tubular damage, highlighting the role of TECs in the pathogenesis of renal disease^[6,7]^. Accordingly, TECs are actively involved in both the acute and chronic phases of kidney disease, making them a promising therapeutic target.

### Pathophysiology of TECs

TECs, particularly those in the proximal tubule, constitute more than 50% of the renal parenchyma^[8]^. These are essential for renal function, playing a pivotal role in the selective reabsorption of water, electrolytes such as sodium and potassium, and nutrients including glucose and amino acids from the glomerular filtrate. Concurrently, TECs actively secrete metabolic waste products, such as urea and creatinine, along with various toxins, into the urine. They also contribute to acid-base homeostasis by reabsorbing bicarbonate and excreting hydrogen ions^[9]^. Owing to their high metabolic activity and energy demands, TECs are particularly susceptible to damage from hypoxia, proteinuria, and toxins.

In response to acute injury, TECs facilitate the regeneration and repair of renal tubules, a process vital for the restoration of kidney function. However, maladaptive repair can result in the AKI-to-CKD transition, culminating in kidney fibrosis^[10]^. Emerging research indicates that TECs are not merely passive targets of injury but also active initiators of the fibrotic response to diverse insults^[11]^. Thus, TECs have been identified as critical therapeutic targets due to their significant involvement in kidney injury and repair mechanisms.

### MSC-EVs

Mesenchymal stromal cells (MSCs), also known as mesenchymal stem cells, are multipotent stromal cells that can be isolated from various adult tissues. They have the capacity to differentiate into multiple cell types, including adipocytes, osteocytes, and chondrocytes^[12]^. Since their first clinical application as a therapeutic agent in 1995^[13]^, MSCs have been considered a promising tool in regenerative and immunomodulatory medicine. Numerous preclinical and clinical studies have demonstrated their potential in kidney protection and repair^[14]^. MSCs exhibit complex and potent endocrine and paracrine functions, with their therapeutic potential largely attributed to their secretome, particularly extracellular vesicles (EVs). Their functions, including growth factor secretion, regulation of inflammation, promotion of cell proliferation, anti-apoptotic and anti-inflammatory effects, reduction of fibrosis, and stimulation of angiogenesis, have been extensively documented in the literature^[15,16]^.

EVs are nanoparticles with a bilayer phospholipid membrane, released by nearly all cell types and commonly detected in various body fluids or cell culture supernatants^[17]^. Based on their biogenesis and size, EVs are generally classified into three subtypes: exosomes (exos), microvesicles (MVs), and apoptotic bodies (ABs), with respective diameters of 30-150 nm, 100-1,000 nm, and 800-5,000 nm^[15]^. Exos (30-150 nm) are generated through inward budding of the cell membrane, forming endocytic vesicles that develop into multivesicular bodies (MVBs). Fusion of MVBs with the plasma membrane leads to the release of intraluminal vesicles into the ECM. Exosomal membranes are enriched with protein markers such as tetraspanins (CD9, CD63, CD81) and heat shock proteins (HSP60, HSP70, HSP90). MVs (150-1,000 nm) are produced by outward budding of the plasma membrane, releasing vesicles that typically contain ADP-ribosylation factor 6 (ARF-6) and mirror the membrane composition of their cells of origin^[18]^. ABs (500 to 2,000 nm) are formed during apoptosis^[19]^. EVs carry a diverse molecular cargo - including nucleic acids, proteins, and lipids - that reflects the physiological state of their parental cells, such as MSCs. This cargo plays a key role in intercellular communication. Owing to their stability and unique biological properties, MSC-derived extracellular vesicles (MSC-EVs) exhibit significant therapeutic potential in kidney disease, while addressing several limitations associated with direct stem cell therapy^[14]^.

Nonetheless, the therapeutic application of MSC-EVs is complicated by their inhernt heterogeneity and the challenges of precise characterization. The biological properties and molecular cargo of MSC-EVs vary considerably, influenced by multiple factors including the tissue origin of the parental MSCs, donor-specific characteristics (such as age and health status), and culture conditions. Variables such as passage number, two-dimensional *vs*. three-dimensional culture systems, oxygen levels, and exposure to inflammatory cytokines all contribute to this variability. Such heterogeneity presents a significant obstacle to the development of standardized therapeutic products^[20]^.

A growing body of evidence from both *in vivo* and *in vitro* studies suggests that MSC-EVs exert protective effects against TEC damage through targeted mechanisms. However, inconsistencies in EV isolation methods and dosing regimens remain a challenge, and it is still unclear whether MSC-EVs act mainly through direct interactions with TECs or indirectly via neighboring cells. In this paper, we critically review the existing literature concerning the targeting of TECs by MSC-EVs in both *in vitro* and *in vivo* settings. We also discuss the limitations and therapeutic potential of MSC-EVs in TEC targeting, with the aim of informing future clinical trials and advancing the clinical application of MSC-EV-based therapies.

## DO MSC-EVs SHOW THERAPEUTIC EFFECTS ON KIDNEY TECs?

### *In vitro* evidence

*In vitro* research has become a widely adopted approach among researchers due to its cost-effectiveness and relative simplicity. TECs, key mediators of kidney fibrosis in response to injury, are increasingly used in *in vitro* studies, particularly in the contexts of AKI, the AKI-to-CKD transition, and kidney fibrosis. Thus, TECs represent a suitable cell type for evaluating the therapeutic efficacy of MSC-EVs *in vitro*. Co-culturing experimentally damaged TECs with MSC-EVs provides a robust platform to examine their direct effects. Multiple studies have demonstrated the therapeutic potential of MSC-EVs for various TEC injuries, as reviewed in detail elsewhere^[21,22]^. To establish TEC injury models, commonly used inducers include cisplatin, lipopolysaccharide (LPS), high glucose, albumin, and transforming growth factor (TGF)-β.

The principal mechanisms through which MSC-EVs confer renoprotective effects include the promotion of cellular proliferation, regulation of apoptosis, autophagy, necroptosis, and inflammatory responses, as well as the mitigation of oxidative stress and cell cycle arrest^[23]^.

These effects are mediated via various signaling pathways and molecular targets. For example, the p53 signaling pathway and caspase activation represent key regulatory mechanisms of apoptosis and cell cycle control^[24]^. In the HK-2 hypoxia model, MSC-EVs were observed to attenuate cell cycle arrest and apoptosis in TECs by downregulating p53 expression and modulating Bcl-2 and Bax, thereby inhibiting apoptosis^[25]^. In a cisplatin-induced HK-2 damage model, EVs derived from MSC-like cells reprogrammed from induced pluripotent stem cells (iMSC-EVs) enhanced cell proliferation and suppressed apoptosis by reversing ERK1/2 pathway activation^[26]^. Similarly, Yin *et al*. reported that bone marrow MSC-derived exosomes (BM-MSC-Ex) inhibit TGF-β1-induced epithelial-mesenchymal transition (EMT) in HK-2 cells through the activation of autophagy^[27]^.

A large body of research has shown that MSC-EVs primarily exert their effects by transferring molecular cargos such as microRNAs (miRNAs), proteins, messenger RNAs (mRNAs), and lipids. Among these, miRNAs have been studied most extensively, with increasing evidence documented in the literature^[25,28,29]^. MiRNAs are small non-coding RNAs, 19-24 nucleotides in length, that regulate up to 30% of mammalian protein-coding genes. Several miRNAs, including miR-125b-5p, miR-20a-5p, and miR-30, have been reviewed for their roles in apoptosis, antioxidation, inflammation, and proliferation^[30]^. In a study by Lindoso *et al*., MSC-EV mechanisms were investigated in an ischemia-reperfusion (I/R) injury model, demonstrating that EV-mediated miRNA transfer and the transcriptional regulation of miRNAs in TECs contributed to protective effects^[31]^. Predicted targets of these miRNAs include genes associated with apoptosis, cytoskeletal reorganization, and hypoxia.

In addition, MSC-EVs can directly deliver various pro-angiogenic transcription factors, including vascular endothelial growth factor (VEGF), insulin-like growth factor 1 (IGF-1), and basic fibroblast growth factor (bFGF), to damaged TECs, thereby promoting angiogenesis - a critical process in tissue regeneration^[32]^. MSC-EVs, particularly MVs, also demonstrate potential in transferring mitochondria and related components in various injury settings. However, their primary role appears to be the modulation of mitochondrial function rather than direct transfer^[33]^.

It is widely accepted that MSC-EVs exert their effects through internalization by TECs, which is typically confirmed using uptake assays. However, comparisons across studies are complicated by differences in experimental design, including incubation times (4-24 h), readout methods (e.g., uptake efficiency or fluorescence intensity), and cell conditions (injured *vs*. normal)^[28,29,34]^. Additionally, critical methodological details, including EV labeling strategies, dye concentrations, removal of unbound dye, and the use of appropriate controls to rule out false positives (e.g., dye aggregation or nonspecific binding), are often inconsistently reported, hindering cross-study comparability. Moreover, uptake assays conducted over 24 h may yield nonspecific signals, potentially resulting in an overestimation of internalization. Importantly, the functional relevance of uptake remains unclear in many studies, as they often do not assess whether internalization translates into the anticipated therapeutic effect in functional assays.

The specific uptake of MSC-EVs by TECs is mediated by ligand-receptor interactions. Lindoso *et al*. showed that MSC-EVs can be internalized by proximal tubular epithelial cells (PTECs) following ATP depletion, mediated via integrins CD29 and CD44 present on the EV membrane. This uptake was inhibited when both integrins were blocked^[31]^. However, the receptors or molecules on PTECs responsible for this interaction were not fully identified. Kidney injury molecule-1 (KIM-1), a marker of tubular damage expressed on injured proximal tubules, also facilitates EV internalization by recognizing phosphatidylserine (PS). Engineered EVs modified with the peptide LTHVVWL, which has a strong affinity for KIM-1, enhanced targeted delivery to injured PTECs^[35]^. Additionally, Cao *et al*. observed that downregulation of ICAM-1 and VCAM-1 in hypoxia/reoxygenation (H/R)-injured HK-2 cells significantly diminished the uptake of Dil-labeled MSC exos^[25]^.

Taken together, current *in vitro* evidence indicates that MSC-EVs exert direct protective effects on damaged TECs, with certain studies showing specific ligand-receptor interactions. However, most *in vitro* studies apply MSC-EVs either as a pre-treatment^[34,36]^ or in co-culture^[24]^, implying that MSC-EVs are introduced prior to the occurrence of damage, which may not accurately reflect *in vivo* conditions. Therefore, it is crucial to further evaluate these effects in animal models and clarify the mechanisms by which MSC-EVs reach the site of injury *in vivo*.

### *In vivo* evidence

*In vivo* animal models provide valuable tools for investigating the functionality of MSC-EVs under pathophysiological conditions, as they preserve intact organ systems and physiological parameters including blood flow and systemic immune responses. Because TECs play crucial roles in both the acute stage (AKI) and the late stage (TIF) of kidney disease, they are commonly used to evaluate the effects of MSC-EVs *in vivo*. Accordingly, MSC-EVs have been tested in various animal models with tubulointerstitial damage, as extensively reviewed in the literature^[22,30,37]^.

Among the various models of kidney damage, cisplatin-induced damage, I/R, and LPS-induced sepsis are frequently employed to investigate AKI, often in conjunction with *in vitro* TEC assays^[38,39]^. By contrast, models such as unilateral ureteral obstruction (UUO), diabetic nephropathy (DN), doxorubicin-induced injury, and adenine-induced injury are more closely related to chronic kidney damage and fibrosis^[40]^. Although animal models cannot fully replicate the complexity of human disease, the careful selection of appropriate models and outcome measures is critical for accurately assessing the effects of MSC-EVs in different contexts. Currently, the anti-apoptotic, pro-angiogenic, anti-inflammatory, and anti-fibrotic properties of MSC-EVs are recognized as key mechanisms underlying their ability to ameliorate kidney damage induced by diverse etiologies^[14,22]^. Furthermore, considering that TECs are rich in mitochondria and highly vulnerable to energy depletion, endpoints such as different types of cell death (apoptosis, autophagy, ferroptosis, pyroptosis), mitochondrial damage, oxidative stress, and inflammation are widely used in *in vivo* studies.

In one study, rats with cisplatin-induced kidney injury were pretreated with human umbilical cord mesenchymal stem cell-derived extracellular vesicles (hucMSC-EVs) via renal capsule injection, which reduced the number of renal tubules exhibiting edema and structural damage. The study further suggested that activation of autophagy is essential for this protective effect, as corroborated by *in vitro* experiments using the rat tubular epithelial cell line (NRK-52E)^[41]^. In another study, Zhao *et al*. demonstrated that in the I/R injury model, MSC-EVs protected TECs by restoring mitochondrial function through mitochondrial transcription factor A (TFAM). TFAM stabilized mitochondrial DNA and suppressed inflammatory responses, resulting in improved serum creatinine and urea nitrogen levels and reduced tubular necrosis^[42]^. In addition, *in vivo* imaging revealed co-localization of Cy7-labeled MSC-EVs with Lotus tetragonolobus lectin (LTL)-positive renal tubules, indicating active uptake of MSC-EVs by TECs. In a separate I/R rat model, administration of adipose tissue-derived MSC-EVs during reperfusion significantly decreased the overproduction of mitochondrial superoxide anion (O_2_•^-^) via activation of the heme oxygenase-1/nuclear factor erythroid 2-related factor 2 (HO-1/Nrf2) antioxidant pathway. However, this intervention did not improve acute tubular injury markers such as KIM-1 and neutrophil gelatinase-associated lipocalin (NGAL) within 24 h. These results highlight both the therapeutic potential of MSC-EVs for early intervention in I/R-induced tubular damage and the importance of selecting appropriate endpoints^[43]^.

Macrophages, as pivotal inflammatory mediators, have also been shown to play a central role in AKI progression^[44]^. In a porcine model of diet-induced metabolic syndrome and renal artery stenosis, Eirin *et al*. demonstrated that MSC-EVs injected into the stenotic renal artery colocalized with tubular cells and macrophages four weeks later. This treatment reduced renal inflammation and stenosis, increased the population of reparative M2 macrophages, and upregulated interleukin-10 (IL-10). Notably, these protective effects were absent when IL-10-depleted MSC-EVs were administered^[45]^. Similarly, in a UUO model, hucMSC-Exos ameliorated renal tubular injury by suppressing macrophage-to-myofibroblast transition (MMT). HucMSC-Exos reduced collagen deposition, lowered tubular injury scores, and downregulated fibrosis markers such as collagen I, vimentin, and α-SMA. Mechanistically, they inhibited MMT by downregulating the circadian gene ARNTL (BMAL1), as evidenced by reduced α-SMA+/F4/80+ dual-positive cells *in vivo* and in TGF-β-induced MMT *in vitro*^[46]^.

Taken together, these studies clearly indicate that MSC-EVs possess therapeutic properties *in vivo* across various models of tubular injury. These effects involve both direct actions on TECs and indirect mechanisms mediated by other cell types, such as macrophages. The kidney, being a complex organ composed of multiple cell types, presents an environment where intercellular interactions can influence the efficacy of MSC-EVs. However, data on the uptake and interaction of MSC-EVs with different nephron cell types remain limited. For instance, damage in one region can exacerbate injury in another by releasing vesicles, as evidenced by injured podocyte-derived EVs inducing apoptosis in TECs^[47]^. Within the nephron system, MSC-EVs can engage with a variety of cell types, including podocytes, TECs, fibroblasts, and macrophages, thereby contributing to renal protection. Consequently, simple *in vitro* co-culture systems involving only MSC-EVs and TECs may not fully capture the complexity of these interactions. More advanced *in vitro* models incorporating multiple cell types will be needed to better elucidate the therapeutic potential of MSC-EVs in the kidney.

## BIODISTRIBUTION AND KIDNEY TARGETING

Despite considerable evidence supporting both the direct and indirect therapeutic effects of MSC-EVs on TECs, it is crucial to investigate their biodistribution in order to clarify the actual fate of MSC-EVs *in vivo*. Such knowledge is fundamental for developing more precise targeting strategies in the future.

### Influence of EV purification, dosage and administration route

Several administration routes have been employed for delivering MSC-EVs in animal studies, including intravenous, intraperitoneal, and *in situ* administration. Regardless of the method used, the *in vivo* biodistribution of injected EVs is most commonly observed in the liver, spleen, lungs, and kidneys^[48]^. This indicates that the injected dose does not necessarily correspond to the amount of EVs reaching the injured site. Increasingly, studies on kidney damage models are incorporating biodistribution analyses or *in vivo* uptake assays. However, significant variability exists across studies with respect to animal species, EV isolation methods, dosages, observation time points, and tracking techniques (e.g., fluorescent dyes, bioluminescence, or radiolabeling).

In studies investigating MSC-EVs for the treatment of kidney disease, rats and mice are the most widely used animal models. The most common administration route is intravenous injection via the tail vein, although some studies employ subcapsular or intrarenal injections to achieve higher kidney retention^[49]^. The isolation method also significantly influences the biodistribution and effective dose of MSC-EVs. Techniques include differential ultracentrifugation (dUC), density gradient separation, size exclusion chromatography (SEC), flow-based separation, and charge- or molecular recognition-based methods^[50]^. These approaches can result in substantial variability in EV purity and cargo composition^[51,52]^. Among them, dUC is the most frequently reported in literature, typically followed by EV quantification based on total protein content measured by standard colorimetric assays. Reported injection doses in rodent models vary widely, from 20 to 200 µg^[53,28]^. Of note, quantification based on total protein may overestimate vesicle dose because of co-isolated protein contaminants, particularly when less specific isolation methods are used^[54]^. Another dosing strategy reported in literature is based on particle number, typically measured by light scattering technologies (such as nanoparticle tracking analysis), with doses ranging from 10^9^ to 10^11^ particles per administration^[43,55]^. Some studies also report doses in terms of the number of producer cells^[56]^. Table 1 summarizes recent research on the biodistribution of intravenously administered MSC-EVs in kidney disease models characterized by tubular damage.

**Table 1:** *In vivo* biodistribution studies of intravenously administered MSC-EVs

**Isolation**	**Injury model**	**EV source**	**Dose**	**Labeling**	**Biodistribution**	**Effects observed**	**Time points**	**Ref.**
dUC	UUO	BM-MSCs	100 μg (mice)	PKH67	Liver, lung, kidneys, brain, spleen, heart; Peak accumulation in obstructed kidney at 72 h	Interstitial fibrosis ↓	6, 12, 24, 72, 168, 240 h	[57]
		hucMSCs	10 mg/kg (rats)	DiR	Liver, lung, kidneys; Enriched in injured kidney	Interstitial fibrosis ↓	48 h	[58]
		AMSCs	1 × 10^3^ μg/mL (mice)	Cy5	Liver, lung, kidneys	Interstitial fibrosis ↓ Peritubular capillaries loss ↓	12 h	[59]
	I/R	hPMSCs	100 μg (mice)	Dil	Injured kidney	Interstitial fibrosis ↓ Mitochondrial FAO ↑ Mitochondrial homeostasis ↑	Day 1, 3, 5, 7	[60]
		BM-MSCs	6.96 × 10^10^ particles (mice)	Sulfo-Cy7-NHS ester	Liver, lung, kidney, spleen; TECs at 24 h	Mitochondrial damage ↓ Inflammation ↓	1, 4, 6, 24 h	[42]
		hucMSCs	100 μg (rats)	PKH-26	Kidney tissue, TECs	Interstitial fibrosis ↓ Angiogenesis ↑ VEGF↑	24 h	[56]
		hPMSCs	80 μg (mice)	AIEgen (DPA-SCP)	Liver, lung, spleen, kidneys; Enriched in injured kidney and TECs	Mitochondrial homeostasis ↑	2, 24, 72 h	[61]
	Diabetic	hucMSCs	10 mg/kg (mice)	Cy7/PKH67	Liver, healthy kidneys, TECs	Apoptosis ↓ EMT ↓	24 h	[62]
	FA-induced CKD	iPSC-MSCs	400 μg (mice)	CellTracker orange CMTMR tetramethylrhodamine	Kidney tissue	Interstitial fibrosis ↓ Inflammation ↓ Cell death ↓	66 h	[63]
TFF	Cisplatin-induced AKI	AMSCs	2 × 10^8^ particles (mice)	Cy5.5	Liver, kidneys; Enriched in injured kidney	Inflammation ↓ Proliferation ↑ Apoptosis ↓	48 h	[64]
AEC	Rhabdomyolysis-induced AKI	iPSC-MSCs	150 μg (mice)	NanoLuc luciferase protein (NanoLuc-sEV)	Brain, lung, heart, liver, spleen, kidneys; Enriched in injured kidney	Inflammation ↓ Proliferation ↑ Klotho loss ↓	1 h	[65]
Commercial kit	UUO	Human MSCs	1 mg/kg (mice)	PKH67	Injured kidney	Interstitial fibrosis ↓	24, 48 h	[66]

↑: Increase; ↓: decrease. dUC: Differential ultracentrifugation; UUO: unilateral ureteral obstruction; I/R: ischemia-reperfusion injury; BM-MSCs: bone marrow-derived mesenchymal stem cells; huc-MSCs: human umbilical cord-derived MSCs; AMSCs: adipose -derived mesenchymal stem cells; hPMSCs: human placenta-derived MSCs; FAO: fatty acid oxidation; TECs: tubular epithelial cells; VEGF: vascular endothelial growth factor; AIEgens: aggregation-induced emission luminogens; EMT: epithelial-to-mesenchymal transition; FA: folic acid; TFF: tangential flow filtration; AKI: acute kidney injury; iPSC-MSCs: induced pluripotent stem cell-derived MSCs; AEC: anion exchange chromatography.

This table highlights a critical barrier: the considerable variability in EV dosage and assessment time points across studies. Even when the same isolation methods are employed, the lack of standardization poses a major obstacle for translating preclinical findings into clinical applications. Another inherent limitation is that most biodistribution studies rely on a single injection, whereas therapeutic studies typically involve multiple administrations. Furthermore, some reports have used the same EV dose for both *in vivo* animal studies and *in vitro* cell experiments, despite these contexts being fundamentally different and not directly comparable^[43,56,61]^. Although a standardized dosing protocol has yet to be established, significant efforts are required to bridge the gap between *in vitro* and *in vivo* research.

### Evidence of MSC-EVs targeting TECs

Existing biodistribution data indicate that MSC-EVs exhibit therapeutic effects on tubular damage. Current research on EV-cell interactions mainly focuses on direct engagement, in which EVs are thought to be internalized by cells or interact with cell surface receptors. To substantiate the hypothesis of direct targeting to TECs, comprehensive *in situ* analyses are required to confirm the presence of MSC-EV within renal tubules. However, their rapid clearance from circulation to organs such as the lungs, spleen, and kidneys within minutes, coupled with challenges in accurate labeling and tracking, poses a significant challenge. Consequently, many studies investigating TECs provide limited evidence of direct targeting, reducing the strength of their conclusions regarding the targeting efficacy of MSC-EVs. Cao *et al*. conducted a real-time *in vivo* tracking study of MSC-EVs in a murine model of I/R injury^[61]^. To facilitate tracking, the researchers labeled MSC-EVs with aggregation-induced emission luminogens (AIEgens), specifically DPA-SCP. A dose of 80 µg of MSC-EVs was administered intravenously, and fluorescence imaging was performed at multiple time points between 2 and 72 h post-injection. The results demonstrated that MSC-EV signals peaked at 12 h and remained detectable for up to 48 h. Furthermore, *ex vivo* tissue analysis combined with LTL staining, a marker of the proximal tubule brush border, confirmed the specific uptake of MSC-EVs by injured TECs. Similarly, another study using the same I/R mouse model detected signals from sulfo-Cy7-NHS ester-labeled EVs in LTL-positive renal tubules at multiple time points following injection^[42]^. However, neither study explored the mechanisms underlying this specific internalization, such as whether it involves ligand-receptor interactions. Elucidating these mechanisms would not only clarify how MSC-EVs target TECs but also inform the development of more effective cell-specific therapies.

### Challenges in targeting: passive vs active approaches

**Figure 1:**
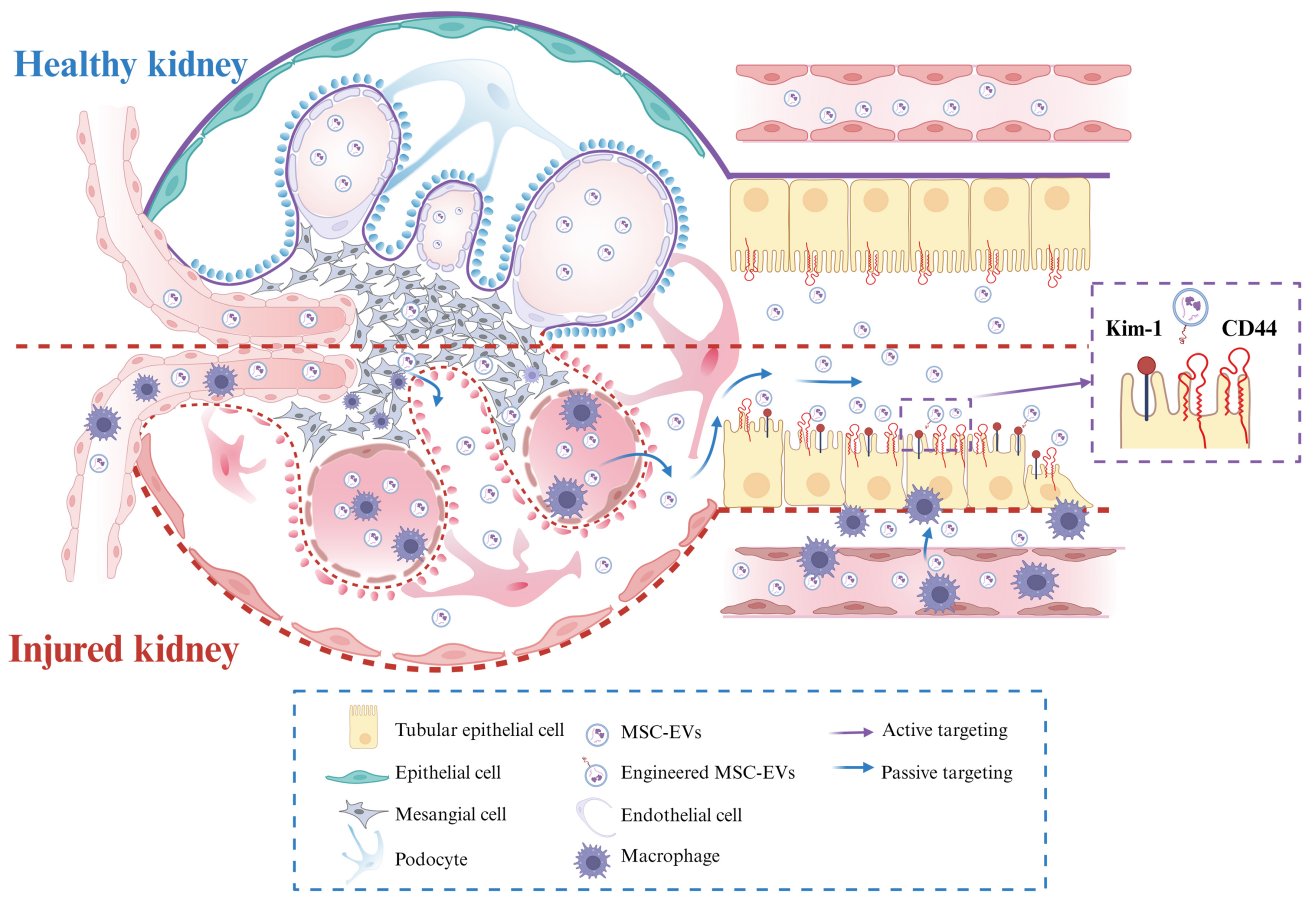
Schematic illustration of MSC-EV delivery and targeting to TECs. Kidney injury can involve disruption of the glomerular filtration barrier, inflammation, immune cell infiltration, and tubular epithelial cell injury. Passive targeting: MSC-EVs can extravasate through a compromised glomerular barrier to reach the tubular lumen or access the interstitial space via injury-induced increases in peritubular capillary permeability. Active targeting: MSC-EVs can be surface-modified for homing to injured TECs using ligands such as CD44-specific antibodies or peptides binding to KIM-1. Created in BioRender. pan, L. (2025) https://BioRender.com/zg9iyea. MSC-EV: Mesenchymal stem cell-derived extracellular vesicle; TEC: tubular epithelial cell; CD44: cluster of differentiation 44; KIM-1: kidney injury molecule-1.

Nanoparticles in circulation, including MSC-EVs, are being investigated for their ability to selectively target TECs through both passive and active mechanisms [Figure 1]. Active targeting relies on specific ligand-receptor interactions between molecules expressed on TECs and MSC-EVs, facilitating therapeutic delivery via receptor-mediated internalization, as discussed in the “*in vitro* effects” section. For instance, the uptake of these vesicles by TECs has been demonstrated *in vitro*, and this process can be inhibited by antibodies against CD44 and CD29 on the MSC-EV membrane^[31]^. Notably, CD44, a ubiquitously expressed type I transmembrane glycoprotein with high affinity for hyaluronic acid (HA), is overexpressed in damaged TECs^[67]^. Engineered EVs derived from PEGylated, HA-modified MSCs selectively accumulate in injured kidneys through HA-mediated binding to CD44 on TECs^[64]^. Similarly, engineering EVs with specific ligands, such as peptides that bind to KIM-1, has been shown to enhance TEC targeting^[35]^.

Passive targeting, conversely, exploits the intrinsic physiological features of the kidney to promote preferential accumulation. Given the complex kidney architecture, which encompasses glomerular filtration and peritubular capillary circulation, nanoparticle size is a key determinant of distribution to TECs. Studies on renal nanomedicine delivery have identified two main theoretical passive pathways: (1) glomerular filtration followed by luminal uptake; and (2) transport through peritubular capillaries followed by basolateral uptake^[68]^.

To reach TECs via glomerular filtration, MSC-EVs must first cross the endothelial cell layer, which is lined with a dense glycocalyx and fenestrations measuring 60-80 nm. Beyond this lies the glomerular basement membrane (GBM), a non-cellular meshwork of negatively charged ECM macromolecules with pores of approximately 10 nm. The GBM is externally covered by podocytes, whose interdigitating foot processes are separated by slit diaphragms averaging 12 nm. These structural constraints suggest that passive diffusion of intact EVs across a healthy GBM is unlikely^[48,69]^. However, under pathological conditions, an impaired GBM with enlarged pores may permit MSC-EV passage, often coinciding with proteinuria. Exploiting such altered barriers, MSC-EVs could potentially traverse the GBM and reach the tubular lumen. Still, whether natural MSC-EVs might also be captured by other injured cells, such as endothelial cells or podocytes, remains unclear. Because glomerular barrier damage is typically accompanied by injury to glomerular cells that can also capture EVs, direct TEC targeting under these conditions becomes more complex. The exact upper size limit for passive passage through a diseased GBM remains to be determined.

Alternatively, MSC-EVs may reach TECs via peritubular capillaries, which lie adjacent to the TEC basal membrane. This anatomical arrangement enables direct transport from the bloodstream into tubular compartments, bypassing glomerular filtration. Although peritubular capillary fenestrae are only ~5 nm, studies using synthetic nanoparticles (e.g., gold or silicon) have shown that particles of 100-200 nm and even 300-400 nm can reach peritubular capillaries and enter TECs through the basolateral membrane via endocytosis and exocytosis^[70]^. Kidney injury, which often occurs in clinical contexts, may further facilitate this process by increasing capillary permeability. Nonetheless, nanoparticle delivery to the tubules is influenced not only by size but also by charge, shape, and nanostructure - critical factors when designing engineered vesicles.

Taken together, future precision medicine strategies employing MSC-EVs to target TECs will likely require an integrated approach, combining passive mechanisms (leveraging nanoparticle size and pathological alterations in diseased kidneys) with active targeting strategies (using TEC-specific ligands) to enhance delivery efficiency while minimizing off-target effects.

## DISCUSSION

Kidney diseases, including AKI and CKD, pose significant global health challenges. TECs play a pivotal role in both the initial injury and the maladaptive repair processes that lead to fibrosis, making them critical targets for therapeutic intervention. MSC-EVs have shown promising renoprotective effects, largely attributable to the bioactive molecules contained within their cargos. This review consolidates current evidence on the mechanisms by which MSC-EVs target and influence TECs, emphasizing both recent advancements and ongoing challenges.

*In vitro* studies provide compelling evidence that MSC-EVs exert direct therapeutic effects on damaged TECs by reducing apoptosis, inflammation, and oxidative stress. These effects are mediated through the transfer of molecules, such as miRNAs and proteins, which modulate key signaling pathways. Importantly, *in vivo* studies indicate that MSC-EVs can actively target TECs through specific ligand-receptor interactions. For example, integrins such as CD29 and CD44 have been implicated in MSC-EV uptake by TECs^[31]^. Additionally, engineering EVs with peptides that bind to markers upregulated on injured TECs, such as KIM-1, enhances targeted delivery^[35]^.

Despite these encouraging results, data remain limited on whether and how MSC-EVs effectively reach TECs *in vivo* - a crucial consideration for designing precise targeting strategies. Biodistribution studies consistently show that systemically administered MSC-EVs predominantly accumulate in organs such as the liver, spleen, lungs, and kidneys, regardless of the administration route. Consequently, only a small proportion of the administered dose reaches the kidneys, and specifically TECs. Some *in vivo* tracking studies demonstrate co-localization of labeled MSC-EVs with renal tubules, suggesting uptake by TECs; however, they also reveal uptake by other cell types, such as macrophages, which contribute to kidney repair. This suggests that the renoprotective effects of MSC-EVs are likely mediated by a coordinated “network-level” modulation involving multiple cell parts and intercellular communication within the injured kidney.

The precise mechanisms by which MSC-EVs reach TECs *in vivo* are not yet fully elucidated. Both passive and active targeting processes are likely involved. Passive targeting is influenced by the physiological and pathological state of the kidney. Two plausible pathways include: (1) glomerular filtration followed by luminal uptake; and (2) access via peritubular capillaries followed by basolateral uptake. Under normal conditions, the glomerular filtration barrier largely restricts EV passage due to their size. While kidney injury may increase pore size, the degree to which intact EVs can traverse this barrier and be selectively internalized by TECs remains unclear. In contrast, peritubular capillary access circumvents the glomerular filtration process, providing direct entry to the basal membrane of TECs. Research involving synthetic nanoparticles indicates that particles of similar size to EVs can reach peritubular capillaries and enter TECs, a process potentially facilitated by increased capillary permeability after injury. In addition to size, EV characteristics such as charge and shape can modulate passive targeting.

A notable limitation in the current literature is the inconsistency in experimental methodologies, especially regarding MSC-EV isolation, characterization, and dosing. Different isolation techniques yield heterogeneous EV populations that are often inadequately defined, with many studies failing to specify the EV subtypes analyzed. Although particle size distributions frequently indicate a predominance of small vesicles, the lack of precise classification complicates dose quantification based on protein content. The wide variation in doses, coupled with the inappropriate application of identical doses for both *in vitro* and *in vivo* studies, impedes meaningful comparisons. Additionally, comprehensive *in vivo* biodistribution studies and high-resolution tracking are often lacking, limiting the ability to definitively confirm TEC-specific uptake and clarify underlying mechanisms. Real-time *in vivo* EV tracking remains a significant technical challenge.

Future research efforts should prioritize enhancing the targeted delivery of MSC-EVs to TECs. This involves optimizing EV characteristics to facilitate passive targeting and, importantly, engineering EVs with ligands that bind to markers upregulated on injured TECs, such as KIM-1 or CD44. Studies must rigorously report methodological details, including EV subtypes, isolation and purification techniques, and comprehensive characterization data. Establishing standardized protocols for EV isolation and quantification is essential to ensure reproducibility. Moreover, advanced *in vivo* biodistribution studies employing high-resolution tracking methods are necessary to verify targeted delivery, quantify the actual dose reaching TECs, and elucidate uptake mechanisms.

## CONCLUSION

MSC-EVs exhibit considerable potential for the treatment of kidney diseases by mitigating TEC damage. This potential is substantiated by robust *in vitro* evidence demonstrating direct protective effects and specific cellular interactions. However, clinical translation is challenged by nonspecific biodistribution and methodological inconsistencies in EV production and characterization. To overcome these obstacles, future research should focus on: (1) standardizing reporting metrics, including particle number, producing cell number, protein content for dosing, and isolation methods; and (2) developing engineered EVs functionalized with ligands targeting markers of injured TECs, such as KIM-1. Combined with advanced *in vivo* tracking techniques, these strategies will be essential for fully realizing the therapeutic potential of MSC-EVs in renal repair.

## DECLARATIONS

### Copyright

© The Author(s) 2025.
